# Pharmacomicrobiomics in Western Medicine and Traditional Chinese Medicine in Type 2 Diabetes

**DOI:** 10.3389/fendo.2022.857090

**Published:** 2022-05-04

**Authors:** Natural Chu, Juliana C. N. Chan, Elaine Chow

**Affiliations:** ^1^ Department of Medicine and Therapeutics, The Chinese University of Hong Kong, Prince of Wales Hospital, Hong Kong, Hong Kong SAR, China; ^2^ Li Ka Shing Institute of Health Sciences, The Chinese University of Hong Kong, Prince of Wales Hospital, Hong Kong, Hong Kong SAR, China; ^3^ Hong Kong Institute of Diabetes and Obesity, The Chinese University of Hong Kong, Prince of Wales Hospital, Hong Kong, Hong Kong SAR, China; ^4^ Phase 1 Clinical Trial Centre, The Chinese University of Hong Kong, Prince of Wales Hospital, Hong Kong, Hong Kong SAR, China

**Keywords:** medication, traditional Chinese medicine, diabetes, microbiota, *Akkermansia*

## Abstract

Pharmacomicrobiomics refers to the interactions between foreign compounds and the gut microbiome resulting in heterogeneous efficacy, side effects, and toxicity of the compound concerned. Glucose lowering drugs reduce blood glucose by modulating insulin secretion and its actions as well as redistributing energy disposal. Apart from genetic, ecological, and lifestyle factors, maintaining an equilibrium of the whole gut microbiome has been shown to improve human health. Microbial fingerprinting using faecal samples indicated an ‘invisible phenotype’ due to different compositions of microbiota which might orchestrate the interactions between patients’ phenotypes and their responses to glucose-lowering drugs. In this article, we summarize the current evidence on differences in composition of gut microbiota between individuals with type 2 diabetes (T2D) and healthy individuals, the disruption of the balance of beneficial and pathogenic microbiota was shown in patients with T2D and how Western Medicine (WM) and Traditional Chinese Medicine (TCM) might re-shape the gut microbiota with benefits to the host immunity and metabolic health. We particularly highlighted the effects of both WM and TCM increase the relative abundance of health promoting bacteria, such as, *Akkermansia muciniphila*, *Blautia, and Bifidobacterium adolescentis*, and which have been implicated in type 2 diabetes (T2D). Several lines of evidence suggested that TCM might complement the efficacy of WM through alteration of microbiota which warrants further investigation in our pursuit of prevention and control of T2D.

## Introduction

Type 2 diabetes (T2D) and its complications constitute a worldwide public health challenge. In 2020, it was estimated that 537 million people had diabetes with the majority residing in low- and middle-income countries (1). Over 95% of affected people have T2D which is associated with an increased risk of premature death and multiple morbidities. Type 2 diabetes is a complex disease due to multiple risk factors where delayed diagnosis and intervention can lead to widespread micro and macrovascular complications. The distribution of gut microbiota in is disrupted in patients with type 2 diabetes and cardiovascular disease. Butyrate producing organisms such as *Bifidobacterium*, *Bacteroides*, *Faecalibacterium*, and *Akkermansia* were negatively associated with T2D, while *Ruminococcus*, *Fusobacterium*, and *Blautia* were more abundant in T2D patients. Gut microbiota can also influence other cardiometabolic risk factors, such as hypercholesteoleremia by modulating metabolite production of bile acids, coprostanol and short chain fatty acids (SCFAs) (2). The balance of beneficial and pathogenic bacteria may linked to different diseases (3). Apart from personal suffering, these complications are associated with enormous healthcare costs and loss of societal productivity, calling for more accurate diagnosis and efficient prevention and control strategies (1).

Rapid urbanization is associated with multidimensional changes in our ecosystem including but is not limited to mechanization, food technology, physical space, cultures, jobs, and leisure which greatly influence our lifestyles notably diet and exercise (4). These changes in the macroenvironment can affect the host internal milieu which can be further influenced by the microorganisms in their gut, referred to as microbiota (2). With the advent of sequencing technology, the collective genomes of these microbiota (microbiome) can be defined and categorized. In recent years, many studies have reported associations of the development of T2D with changes in the gut microbiome (5, 6). Possible mechanisms include insulin resistance, changes in pH and bowel permeability (7), endotoxemia (8) as well as changes in the metabolism of bile acids (9) and short-chain fatty acids (SCFA) (10, 11).

In support of the possible causative roles of microbiota in the pathogenesis of T2D, there are also reports suggesting that some Western Medicine (WM) and Traditional Chinese medicine (TCM) mediated their effects through changes in the microbiome (12). In this review, we summarize differences in the composition of gut microbiota between healthy individuals and patients with T2D as well as the effects of different WM and TCM on gut microbiota, which act in concert with lifestyle factors to orchestrate the diversity of the whole gut microbiome and influence metabolic health.

## Differences of Gut Microbiota in Healthy Individuals and Patients With T2D

Microbial fingerprinting refers to the use of fecal samples to identify the unique pattern of the microbiome, referred to as ‘dysbiosis’, associated with a disease phenotype. In this section, we reviewed published data on the pattern of microbiota in T2D and explored the possible effects of different medications in altering microbiota homeostasis.

In a recent systematic review, patients with T2D had a higher abundance of *Lactobacilli* and a lower abundance of *Bifidobacteria* than healthy individuals (5). In this analysis which included 13 case-control studies including 575 patients with T2D and 840 healthy controls, the authors reported that these T2D-associated microbiome might be further influenced by the effect of different medications. In another study involving 11 newly diagnosed patients with T2D, researchers compared their microbiota with that of 17 individuals with prediabetes and 39 patients with established T2D. Compared to healthy individuals, newly diagnosed T2D had a lower abundance of *Akkermansia*, *Blautia*, *Ruminococcus* (13),*Clostridium leptum, and Clostridium coccoide (* 14), but these changes were restored in patients with T2D on antidiabetic treatment (15). *C. leptum* and *C. coccoides* are butyrate-producing bacteria and are inversely related to glucose and homeostatic model assessment (14).

Several lines of evidence indicated that intestinal microbial overgrowth was found in patients with newly diagnosed T2D compared with individuals with normal glucose tolerance (NGT). While individuals with impaired glucose tolerance (IGT) and T2D had similar patterns of dysbiosis, this was not found in those with impaired fasting glucose (IFG) (16). In a 4-year study involving individuals with prediabetes, researchers reported plasma glucose was negatively associated with *Odoribacter*, *Oscillibacter*, and *Pseudoflavonifracter* (15).

*Clostridium leptum* and *C. coccoides* were microbiota that could influence human health by altering intestinal peristalsis, promoting synthesis of vitamins, promoting excretion of harmful substances, and protecting the gut from an invasion of pathogens. In treatment-naïve patients with T2D, there was relative depletion of *C. coccoides* and *C. leptum* considered to be health-promoting microbiota. In these patients, the microbiota was also dominated by harmful microbiota, such as *Escherichia/Shigella* (17). Other species implicated in T2D included *Akkermansia, Blautia, Clostridium* spp., and *Ruminococcus.* Of note, low abundance of *Akkermansia muciniphila* had been associated with obesity and aging while its administration had been shown to increase the intestinal levels of endocannabinoids with reduced inflammation (18). Recently, some species in the genera *Clostridium* and *Ruminococcus* had been reclassified as *Blautia*, the latter being a newly discovered anaerobic probiotic which was negatively correlated with metabolic diseases such as T2D, obesity, and fatty liver (19). All these studies found a decrease in the number of butyrate-producing bacteria, such as *Akkermansia, Blautia*, and *Bifidobacteria*, and an increase in conditional pathogens, *Escherichia/Shigella.*

## Effects of WM on the Gut Microbiome in Type 2 Diabetes

**Table 1** summarizes the effects of WM on the composition of the microbiota. Biguanide (e.g., metformin) is the most popular oral glucose-lowering drug often used as first-line therapy in patients with T2D. Metformin has pluripotent effects which improve energy metabolism and reduce inflammation. By inhibiting the mitochondrial complex I as a key component of the electron transport system, metformin activates AMPK (adenosine 5′- monophosphate-activated protein kinase) resulting in reduced anabolism and increased catabolism (27). Metformin also reduces hepatic glucose production and absorption of glucose from the intestine. Additionally, the gut microbiota has been linked to the glucose-lowering efficacy and tolerance with metformin.

**Table 1:** Summary of the effects of western medicine on the composition of the gut microbiome in T2D patients.

Drugs	Author (Years)	Patients	Periods	Study design	Effect of treatment on microbes	Additional remarks
Metformin	Wang et al., 2018 (20)	37 patients with T2D (18 treated with metformin and 19 treated with GLP-1 mimetics)	6 weeks	Cross sectional study	Metformin *↑Sutterella*	A higher abundance of *Akkermansia* in patients with short and medium duration than those with long duration of diabetes
Metformin	Sun et al., 2018 (11)	22 patients with newly diagnosed T2D treated with metformin	3 days	Intervention study	Metformin *↑Lactobacillus sanfranciscensis* ↓*Bacteroides fragilis*	Metformin improves obesity-induced glucose intolerance and insulin resistance through the gut microbiota
Metformin	Wu et al., 2017 (21)	40 patients with newly diagnosed T2D treated with metformin	4-6 months	Randomized placebo controlled crossover study	Metformin *↑Akkermansia muciniphila, Bifidobacterium adolescentis, Lactobacillus fermentium, Peptoniphilus* sp. *Ruminococcus* sp.*, etc. ↓Intesinibacter bartlettii, and Clostridium* spp.	Decrease in HbA1c and fasting plasma glucose after the metformin treatment
Metformin	Cuesta-Zuluaga et al., 2017 (22)	28 patients with T2D (14 treated with metformin and 14 not-treated with metformin) and 84 without diabetes	Not mentioned	Cross-sectional case-control study	Metformin *↑Akkermansia muciniphila, Butyrivibrio, Bifidobacterium bifidum, Megasphaera*, and *Prevotella*	There were significant differences in the comparison in β diversity of microbiome between metformin and non-metformin users
Acarbose	Gu et al., 2017 (23)	94 patients with newly diagnosed T2D treated with acarbose or glipizide	3 months	Multicentre parallel comparison	Acarbose *↑ Lactobacillus* and *Bifidobacterium ↓Bacteroides*	Reductions in HbA1c, fasting, and postprandial plasma glucose in both treatment arms
Acarbose	Su et al., 2015 (24)	59 patients with T2D patients treated with acarbose 36 patients treated with other glucose-lowering drugs 55 healthy controls	4 weeks	Cross-sectional case-control study	Acarbose *↑Bifidobacterium longum*	Acarbose significantly reduced lipopolysaccharides and prothrombin activator inhibitor-1
GLP-1 mimetics	Shang et al., 2021 (25)	40 patients with T2D switched from metformin to liraglutide	4 months	Observational study	Liraglutide *↑Collinsella*, *Akkermansia*, and *Clostridium*	BMI, HbA1c, homeostasis model assessment of insulin resistance (HOMA-IR), fasting blood glucose, 2-hour postprandial blood glucose, and lipid profiles were significantly lower after liraglutide-treatment
GLP-1 mimetics	Wang et al., 2018 (20)	37 patients with T2D (18 treated with metformin and 19 treated with GLP-1 mimetics)	6 weeks	Cross-sectional study	GLP1 *↑Akkermansia*	Patients receiving a GLP-1 agonist had higher *Akkermansia* abundances than those on metformin.
SGLT2i and Gliclazide	Bommel et al., 2019 (26)	44 metformin-treated patients with T2D randomized to either dapagliflozin or gliclazide	12 weeks	Randomized double-blind, comparator-controlled, parallel-group trial	No change in microbiota with either dapagliflozin or gliclazide treatment	Both drugs improved glycaemic control with dapagliflozin reducing and gliclazide increasing fasting plasma insulin.

GLP-1, Glucagon-like peptide-1.

In an animal study, 28 high-fat-fed mice were randomized to the control and metformin group equally, metformin group increased the abundance of genus *Bacteroides, Akkermansia, Parabacteroides, Christensenella, Clostridales*, and decreased the abundance of genus *Muribaculum, Lachnoclostridium, Coprococcus, Dorea, Papillibacter, Oscillospira, Ruminococcus*, and *Desulfovibrio* in 12 week treatment (28). In other animal studies, metformin has been shown to constantly promote of the abundance of *Akkermansia* at a dose ranging from 75 to 300mg/kg/d given for 4 days to 14 weeks in ten controlled studies (29–38). In a 4-month double-blind, placebo-controlled study involving 40 patients with T2D, who were treated with metformin showed no differences in body weight, body fat, and fasting plasma insulin but reduced glycated hemoglobin (HbA1c) and fasting plasma glucose. Treatment with metformin also increased *Akkermansia muciniphila*, *Bifidobacterium adolescentis, Lactobacillus fermentium, Peptoniphilus* sp. *Ruminococcus* sp. *Cronobacter turicensis, Enterobacter lignolyticus, Citrobacter koseri, Yersinia enterocolitica subsp., Klebsiella pneumonia, Enterobacter asburiae, Enterobacter cloacae subsp*, and decreased *Intestinibacter bartlettii, Clostridium beijerinckii, Clostridium* sp. *Clostridium perfringens, Clostridium botulinum*, and *Clostridium butyricum*. Notably, *Bifidobacterium adolescentis* was the only probiotic that exhibited a dose-related response to metformin in the gut microbiome. In animal and human studies, metformin increased the abundance of *Akkermansia muciniphila* (39) but inconsistent in other health promoting microbiota, such as *Blautia (* 40), *Prevotella (* 41), and *Roseburia* (38). The results in humans are different from the results of animal studies because of differences in intestinal microbiome between humans and animals (42) affecting by eating habit, physical activities, ethnic origins, course of disease, comorbidities, and multiple medications.

In another two clinical trials, metformin also increased *Akkermansia muciniphila* and SCFA-producing microbes (10) including *Butyrivibrio, Bifidobacterium bifidum, Megasphaera*, and *Prevotella* (22). These microbes utilized multiple dietary substrates to produce an array of metabolites. The abundance of *Bifidobacterium* species can activate multiple genes involved in carbohydrate metabolism (43) and *Prevotella* species contribute to starch degradation (22). In another study involving patients with newly diagnosed T2D, 3-day treatment with metformin decreased the genus *Bacteroides* with increased bile acid glycoursodeoxycholic acid (GUDCA) accompanied by reduced hyperglycemia. In mice, colonization of *B. fragilis* abrogated the glucose-lowering and GUDCA increasing effects of metformin suggesting that this microbe might play a mediating role in these metabolic effects of metformin (11).

Acarbose is an alpha-glucosidase inhibitor. It is a highly popular glucose-lowering drug in China (44) and many Asian countries (45). This WM is a complex molecule that inhibits the conversion of disaccharides to monosaccharides and thus converts carbohydrates into a fiber-like molecule. This leads to an increased amount of indigestible carbohydrates in the lower part of the intestine available for fermentation by microbiota. In an animal study, compared to the control and a low dose acarbose (25 ppm), a high dose of acarbose (400 ppm) promoted the abundance of *Bacteroidaceae* and *Bifidobacteriaceae* and decreased in the abundance of *Bacteroidales S24-7* and species *Akkermansia muciniphila* under the controlled diet (46). However, in another animal study when compared to placebo, acarbose displayed a higher abundance of *Ruminococcus 2* and *Lactobacillus* and decrease the species *Akkermansia muciniphila (* 47). In a human study, after treatment with acarbose, *Lactobacillus* and *Bifidobacterium* species thrived with depletion of the original gut microbiota including *Bacteroides*, *Alistipes*, and *Clostridium* (23). In a clinical study, acarbose was found to increase the abundance of *Bifidobacterium* and *Lactobacillus*, which correlated inversely with changes in HbA1c and body weight. At the genus level, acarbose decreased the abundance of *Bacteroides* and at a species level, *Bacteroides plebeius, Bacteroides dorei/vulgatus*, and *Clostridium bolteae*. In a randomized trial, acarbose treatment increased the abundance of *Bifidobacterium longum* and *Enterococcus faecalis* in patients with T2D. However, these results might have been confounded by its co-administration with metformin and other glucose-lowering drugs (24) which could also alter the diversity of microbiota. In people with prediabetes (48), compared to placebo, treatment with acarbose increased abundance of *Lactobacillus* and *Dialister* and reduced abundance of *Butyricicoccus*, *Phascolarctobacterium*, and *Ruminococcus.* However, the study did not differentiate between IFG and IGT. Such differentiation is important given that the microbiome in the IGT group is more akin to that in individuals with T2D (16).

Dipeptidyl peptidase (DPP-4) inhibitors include sitagliptin, saxagliptin, linagliptin, and alogliptin. This drug class prevents the enzymatic degradation of glucagon-like peptide 1 (GLP-1) and glucagon-like peptide 2 (GLP-2). Glucagon-like peptide (GLP) and glucose-dependent insulinotropic polypeptide (GIP) are incretins or peptides secreted by the enterocytes in the gut. Incretins are natural hormones that suppress glucagon and hepatic glucose production whilst augmenting insulin secretion during meal time resulting in reduced fasting and post-prandial blood glucose. In animal studies, DPP4-inhibitors reduced the abundance and diversity of gut microbiota accompanied by reduced body weight (49). In one animal study, DPP-4 inhibitor vildagliptin decreased *Oscillibacter* spp. and increased *Lactobacillus* spp (50). In mice treated with sitagliptin; one-third of the total species were occupied by *Ruminococcaceae*. These results suggested that sitagliptin might alter the gut microbiome to promote fermentation of complex plant-based carbohydrates and influence host metabolism (51). Other animal studies also showed that DPP-4 inhibitor increased the abundance of *Roseburia* and decreased *Blautia* with no effect on *Clostridium* (52).

In animal studies, GLP-1 receptor agonists reduced hyperglycaemia which was associated with a reduced abundance of *Romboutsia* and *Ruminiclostridium* as well as an increased abundance of *Prevotella* was associated with reduction of body weight (53). Neither GLP-1 receptor agonists nor DPP-4 inhibitors induced diversity of microbiome when used as an add-on therapy to metformin or sulphonylureas (SU) in the human study (54). Since metformin might have reshaped the microbiota, there might be little room for further changes by GLP-1 and DPP-4 inhibitors. However, in another clinical study involving 40 patients with T2D who were switched from metformin monotherapy to daily subcutaneous liraglutide injection for 4 months, there was an increase in the abundance of *Collinsella*, *Akkermansia*, and *Clostridium* genus (25).

Sodium–glucose-transporter-2 (SGLT2) inhibitors increase urinary glucose and sodium excretion resulting in a reduction in blood glucose, plasma insulin, blood pressure, and body weight (55). In the animal study, dapagliflozin, a SGLT2 inhibitor reduced the *Firmicutes* to *Bacteroidetes* ratio and increased the abundance of *Akkermansia muciniphila* (39). However, in a subsequent double-blind, randomized clinical trial comparing dapagliflozin and gliclazide, the latter being a sulphonylureas, in patients with T2D (26), neither drug induced any changes in the composition of gut microbiota. Sulphonylureas reduce blood glucose by directly stimulating insulin secretion. In a clinical trial, gliclazide, a sulphonylureas, did not change the relative abundances of microbiota (23). In a 3-month observational study, another sulphonylureas, glipizide, also did not cause changes in the microbiota (23). In these clinical studies, the majority of patients were treated with metformin which was well known to alter the relative abundance of microbiota. Thus, the addition of dapagliflozin and gliclazide as add-on medications might not induce further significant effects. Besides, given their mechanisms of action which are largely independent of the gut, the neutral effects of SGLT2 inhibitors and sulphonylureas are not unexpected. However, most of these results in different WMs were not consistent due to the complex composition of the microbiota, the large variation between individuals in different cultures, and the differences in experimental design affecting by the effect of multi-therapy in treatment of WMs in human gut microbiota. Taken together, the conduct of well-designed, double-blind, placebo-controlled studies preferably in newly diagnosed, treatment-naïve patients with T2D and prediabetes are needed to clarify the mediating effects of microbiota on WM in influencing metabolic health.

## Effects of TCM on the Gut Microbiome in T2D

Due to potential side effects of WM, notably hypoglycemia, as well as for reasons such as cultures, traditions, and social norms, TCM has always been an integral part of clinical practices and therapeutics in East Asian countries. Similar to metformin and acarbose, there is emerging evidence suggesting that TCM might alter the diversity of the gut microbiome with alteration of bile acid metabolism and increased production of SCFAs which contribute to the improvement of glucose metabolism. Herein, bile acids are cholesterol-derived metabolites that promote the intestinal absorption and transport of dietary lipids and play a key role in energy metabolism (56). **Table 2** summarise the effects of TCM on microbiota and metabolic effects.

**Table 2:** Summary of the effects of traditional Chinese medicine on the composition of the gut microbiome in T2D patients.

TCM	Year	Patients	Period	Study design	Microbes	Outcomes
Berberine	Zhang et al., 2020 (57)	409 Patients with T2D treated with either berberine alone, probiotic+berberine, probiotic alone or placebo.	12 weeks	Randomized, double-blind, placebo-controlled trial	Berberine ↓ *Ruminococcus bromi*	Berberine reduced HbA1c, fasting and postprandial plasma glucose, fasting plasma triglyceride, total and low-density lipoprotein cholesterol
GQD	Xu et al., 2015 (58)	187 patients with T2D treated with either GQD or placebo	12 weeks	Randomized double-blinded placebo-controlled clinical trial	GQD ↑ *Faecalibacterium prausnitzii*	GQD reduced the mean fasting plasma glucose and HbA1c
AMC	Tong et al., 2018 (59)	100 patients with T2D treated with either the metformin or AMC	12 weeks	Randomized, open labelled randomized study RCT	AMC ↑ *Faecalibacterium* spp.	AMC reduced plasma glucose and lipids

GQD, Gegen Qinlian Decoction; AMC, specifically designed herbal formula (no full name provided).

Berberine is the main ingredient of TCM used for treating T2D. It is a natural plant alkaloid extracted from *Berberis aristata* and *Coptis chinensis* (Huanglian) (60). Berberine has reduced solubility in the gut and can permeate the gut wall. In a 12-week randomized clinical trial comparing berberine and placebo, berberine altered the gut microbiome composition with a 2-fold increase in *Bacteroides* spp. and *Proteobacteria* (61), a pattern similar to that due to metformin (11, 21). Berberine also induced cell death in harmful gut bacteria and enhanced the composition of beneficial bacteria including *Bifidobacterium adolescentis* and *Lactobacillus acidophilus* (61). Both berberine and metformin upregulated the AMPK pathway which reduced anabolism and promoted catabolism including glycolysis resulting in weight loss and reduced insulin resistance (62). Despite these beneficial effects, berberine depleted the SCFA- producing microbes including *Roseburia* spp.*, Ruminococcus bromii, Faecalibacterium prausnitzii*, and *Bifidobacterium* spp. These two species (*Roseburia* spp. *and Bifidobacterium* spp.) are biomarkers indicative of a healthy gut microenvironment. Other researchers reported an inverse association of *Ruminococcus bromii* with bile acid metabolism with reduced formation of secondary bile acids by microbiota (57). In the intestine, bile acids undergo multistep biotransformation catalyzed by enzyme activities in gut bacteria, and the increase of *Ruminococcus bromii* suppresses bacterial 7α-dehydroxylase and leads to the reduction of secondary bile acids (63, 64). Although berberine lack some of the favourable effects of metformin on microbiota, it possessed beneficial effects exhibited by acarbose treatment.

Gegen Qinlian Decoction (GQD) is another popular TCM for the treatment of T2D. It comprises seven herbs including *Rhizoma coptidis*, *Radix scutellariae, Radix puerariae*, *Rhizoma anemarrhenae*, *Radix panacis uinquefolia*, *Radix paeoniae rubra* and *Rhizoma zingiberis* (65). The effects of GQD on microbiota were similar to that of berberine. GQD treatment altered the overall gut microbiota structure and enriched many butyrate-producing bacteria, including *Faecalibacterium*, *Bifidobacterium*, and *Gemmiger.* These changes in the gut milieu had been shown to attenuate intestinal inflammation and improve metabolic health including glucose metabolism. In the animal study, both berberine and GOD increased the plasma levels of SCFA with reduced fasting plasma insulin level (58). In another study, treatment with GQD enriched the abundance of Faecalibacterium prausnitzii which was negatively correlated with fasting and 2-hour postprandial blood glucose and HbA1c as well as positively with insulin response as indicated by the HOMA-β index (66). One of the ingredients in GQD, *Radix scutellariae*, is commonly co-investigated with metformin (67). TNF-α was significantly reduced and the abundance of *Lactobacillus* and *Akkermansia* remarkably increased after metformin treatment with *Scutellaria baicalensis* when compared to metformin treatment with placebo.

JinQi Jiangtang (JQJT) is a formula used for the prevention of T2D. It contains *Rhizoma coptidis, Astragali Radix and Lonicerae Japnicae Flos.* In the animal study, treatment with JQJT tablets increased the abundance of species *Akkermansia* and reduced that of genus *Desulfovibrio*. Of note, reduced abundance of *Akkermansia spp* was correlated with inflammation in people with obesity (68). Other studies had reported that JQJT tablets modulated gut microbiota with increased formation SCFAs. The latter can provide energy and nutrition for the intestinal epithelium with improved gut health (69). There are limited studies on the effects of JQJT on microbiota in patients with T2D. In a 2-year multi-center randomized clinical trial involving 400 Chinese individuals with prediabetes, treatment with JQJT was associated with a lower incidence of diabetes compared to placebo with reduced blood glucose, triglyceride, albuminuria, and insulin resistance although there was no information on microbiota (70).

A modern herbal formula called AMC (no full name provided in the article) had been specifically developed for the treatment of T2D with hyperlipidemia. The herbs used in this formula included *Rhizoma Anemarrhenae, Momordica charantia, Coptis chinensis, Aloe vera*, and red yeast rice. In a randomized study comparing AMC and metformin in patients with T2D, AMC was similarly efficacious as metformin in reducing blood glucose and lipid levels. Both metformin and AMC enrich the abundance of beneficial bacteria *Blautia* spp., which correlated with improvements in glucose and lipid homeostasis. However, AMC showed better efficacy than metformin in improving HOMA-IR and plasma triglyceride *via* an increase of *Roseburia*, *Faecalibacterium, Gemmiger, Coprococcus*, and *un-Lachnospiraceae* (59).

## Interaction Between WM and TCM

**Figure 1:**
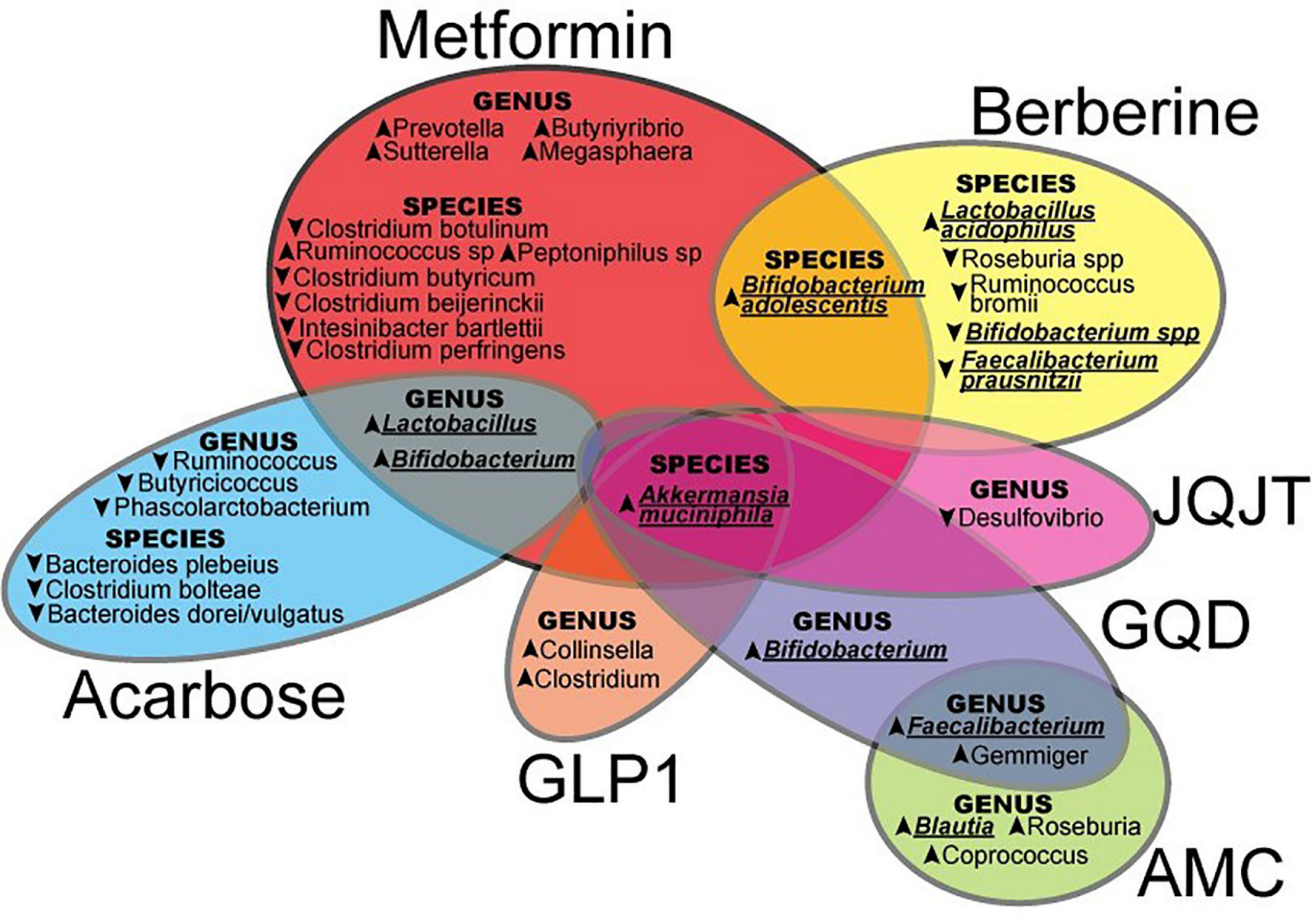
The effects of Western Medicine and Traditional Chinese Medicine in shaping the gut microbiota which may contribute to the control and prevention of type 2 diabetes (JQJT, JinQi Jiangtang; GQD, Gegen Qinlian Decoction; AMC, specifically designed herbal formula (no full name provided); GLP-1, Glucagon-like peptide-1.) In this review, summarized evidence suggested that both WM and TCM orchestrated different patterns on the microbiome, upward and downward arrows indicated an increase or decrease of certain microbiota by WM or TCM, and the particular microbiota underlined were possibly highlighted in the treatment of T2D.

Current evidence suggested that both WM and TCM orchestrated different effects on the microbiome (**Figure 1**) to modulate glucose metabolism through different mechanisms. In East Asia with a large number of people with T2D, herbal medicines are frequently used as complementary therapies by patients treated with WM, notably metformin, although co-administration of TCM and WM is lacking in the clinical guideline. Few studies evaluated possible WM-TCM interactions including pharmacokinetics and pharmacodynamics. In recent human study, compared with administration of metformin and placebo, co-administration of metformin and berberine resulted in significant improvements in glycemic control, liver fat content, and body weight (71). In an animal study, compared with administration of metformin alone, co-administration of berberine and metformin resulted in changes in the gut microbiome due to reduced metformin degradation. These changes included an increased abundance of *Bacteroides fragilis*, *Clostridium perfringens*, *Staphylococcus aureus*, *Klebsiella pneumoniae*, *Escherichia coli*, and *Enterobacter cloacae*, which might adversely affect the host immunity. These less desirable changes suggested berberine might attenuate the favourable effects of metformin on microbiota (72). Further investigations are warranted to evaluate the impacts of WM-TCM interactions on microbiota and human health.

## Other Factors Affecting Drug-Microbiome Interactions

Host genomes, dietary habits, and physical activities are the most important factors that might confound drug-microbiome interactions. Within the same population, researchers reported considerable inter-individual as well as intra-individual variations in their microbiome patterns such as the ratio of *Firmicutes* to *Bacteroidetes* which are the two major phyla in the gut (73). These differences are most likely due to differences in dietary habits, physical activity, and consumption of different drugs such as antibiotics.

### Dietary Factors and Physical Activities

Dietary factor is directly interacting with gut microbiota and many research had indicated that different diets orchestrate the pattern of microbiota (74–76). Other researchers had reported that habitual dietary consumption caused changes in the composition of gut microbiota which in turn influenced the effects of their drug therapy. In Japanese patients with T2D taking acarbose, high rice intake was associated with the abundance of *Faecalibacterium* while high intake of potatoes was associated with a low abundance of health-promoting microbiota such as *Akkermansia* and *Subdoligranulum* (77). In another human study, compared to 20 obese women before metformin treatment, an increase of *Escherichia/Shigella* was found after 2 months of low-calorie diet and metformin treatment (78). This results did not suggest in other similar study design of human (22) and animal (79) studies when having metformin alone. These findings lent support to the hypothesis that diet-drug interaction may alter the microbiota to either attenuate or augment the therapeutic efficacy of WM or TCM.

In a human study, 26 subject sedentary lifestyle and prediabetes or T2D were increased exercise for 2 weeks, a decrease in the *Clostridium* genus was observed (8). In another study, 12-week intense exercise-induced changes in the gut microbiota in subjects with prediabetes with marked improvement in insulin resistance and reduced insulin level. This was accompanied by decreases in *Bacteroides xylanisolvens* and an increase in the abundance of *Streptococcus mitis* (80). However, no study investigated the effect of medicine and exercise on the gut microbiota in T2D.

## Drug-Gut-Microbiota Cross-Talks and Drug Efficacy

Many oral glucose-lowering drugs might cause gastrointestinal side effects, partly due to fermentation of undigested carbohydrates by microbiota resulting in gas formation with altered transit time and gut permeability. These side effects might be alleviated using prebiotics or probiotics to improve treatment tolerance and glycemic control. Prebiotics and probiotics are microbiota-management tools for improving host health. Prebiotics are a group of nutrients in natural foods that are selectively utilized by host microorganisms conferring a health benefit and probiotics are health-related microbial strains and act as an oral supplement or added into food products (81). In a clinical study involving ten metformin-intolerant patients with T2D, administration of a readily dissolvable powder containing inulin, beta-glucan and polyphenols modulated the microbiome with improved metformin tolerance (82). Inulin and beta-glucan are metabolized in the colon by *Bacteroides* and *Prevotella* genera (83) with increased secretion of peptide YY and GLP-1. These changes were accompanied by reduced fasting plasma glucose and frequency of loose stool, a common side effect of metformin.

Since orally administered drugs may shape the gut microbiota, researchers suggested that probiotics might be used as an adjunctive to WM aimed at altering the diversity of microbiota with increasing SCFAs and enhanced glucose management. In a randomized placebo-controlled study, co-administration of probiotics (*Lactobacillus* spp.*, Bifidobacterium* spp.*, Streptococcus* spp., and *Saccharomyces* spp.) in 60 subjects with prediabetes or T2D, did not improve glycemic control but increased insulin sensitivity. There was an increase in the relative abundance of *Bifidobacterium breve* and *Akkermansia muciniphila* and *Clostridium XIVa*, albeit short of significance compared with the placebo group (84). Whether administration of prebiotics to augment the health-promoting effects of microbiota might be more effective than direct administration of health-promoting probiotics in improving drug tolerance or metabolic health is a subject that warrants further investigations.

### Future Perspectives

Much remains unknown on the effect of glucose-lowering WM and TCM on microbial composition and interaction with host factors. In addition to effects on blood glucose, changes in microbiota may also improve other cardiometabolic risk factors. Modulation of the microbiota be part of a new therapeutic strategy against other diseases, such as non-alcoholic fatty liver disease (85), cardiovascular or even neurodegenerative disorders (86). For example, *Akkermanisa* spp., which is increased by metformin, was also highly correlated with weight loss (74). There is emerging evidence for a pro-inflammatory dysbiosis in neurodegenerative disorders such as Parkinson’s disease. The decrease in anti-inflammatory genera such as *Blautia*, *Coprococcus*, *Roseburia*, and *Fecalibacterium (* 87 *)*, could potentially be reversed by metformin or acarbose. Finally, pharmacomicrobiomics should evaluate interactive effects between WM and TCM in the treatment of diabetes, where either beneficial or harmful drug interactions mediated *via* microbiota might occur.

## Conclusion

Type 2 diabetes is a disorder of energy metabolism due to complex interplays amongst the ecosystem, host, and microbiome. The natural history of obesity, prediabetes and diabetes are associated with inter-individual and intra-individual diversity of microbiota. Diabetes-associated dysbiosis is characterized by a reduction in gram-positive members of the beneficial microbiota such as *Blautia*, *Rumminococcaceae*, and gram-negative *Akkermanisa* species with reduced production of SCFA and dysregulation of bile acid metabolism which can adversely affect metabolic health.

Glucose lowering drugs can alter glucose, lipid, and fat metabolism and modulate inflammatory responses by re-shaping the composition of the microbiome which in turn can affect immune cells directly and indirectly through metabolites such as lipopolysaccharide and SCFAs, alteration of gut permeability, and whole gut transit time. Host-gut microbiota interaction is central in bile acid metabolism and cell signalling and can be modulated by medications. The effects of these changes in gut microbiota might contribute to the diversity in disease phenotypes including hormones and inflammatory cytokines. Both WM (e.g. metformin and acarbose) and TCM (berberine based) have been shown to improve the abundance of beneficial bacteria, such as *Blautia* spp.*, Akkermanisa* spp., *and Faecalibacterium*, and reduce the production of secondary bile acids which might contribute towards their metabolic effects including their side effects. Integration of WM and TCM may promote different health-related microbiota and suppress the pathogenic microbiota, such as *Desulfovibrio.* Given the expanding knowledge in the field of microbiome and the availability of high throughput sequencing, further investigations on the modulating effects of microbiota on the efficacy and side effects of WM and TCM will provide novel insights and open a new avenue for reducing the burden of T2D and non-communicable diseases.

## Author Contributions

NC and EC conceived of the presented idea. NC drafted the manuscript and designed the figures. JC and EC devised the main conceptual ideas and proof outline. JC encouraged NC to investigate the Chinese Medicine and supervised the findings of this work. All authors discussed the results and contributed to the final manuscript.

## Conflict of Interest

The authors declare that the research was conducted in the absence of any commercial or financial relationships that could be construed as a potential conflict of interest.

## Publisher’s Note

All claims expressed in this article are solely those of the authors and do not necessarily represent those of their affiliated organizations, or those of the publisher, the editors and the reviewers. Any product that may be evaluated in this article, or claim that may be made by its manufacturer, is not guaranteed or endorsed by the publisher.
